# Interfacial Bonding and Fracture Behaviors of AZ63 Magnesium Alloy Sheet Processed by Accumulative Roll Bonding

**DOI:** 10.3390/ma16144981

**Published:** 2023-07-13

**Authors:** Junqing Guo, Wanting Sun, Nan Xiang, Fuxiao Chen

**Affiliations:** 1 School of Materials Science and Engineering, Henan University of Science and Technology, Luoyang 471023, China; hkdgjq@163.com (J.G.); xiangnan@haust.edu.cn (N.X.); 2 Department of Industrial and Systems Engineering, The Hong Kong Polytechnic University, Hung Hom, Hong Kong 100872, China; 3 Provincial and Ministerial Co-Construction of Collaborative Innovation Center for Non-Ferrous Metal New Materials and Advanced Processing Technology, Luoyang 471023, China; fxchen@haust.edu.cn

**Keywords:** accumulative roll bonding, magnesium alloy, microstructure evolution, interfacial bonding, fracture behavior

## Abstract

In order to understand the strengthening and the failure mechanism of accumulative roll bonding (ARB)-processed AZ63 Mg alloy, the interfacial bonding and fracture behavior of an ARB-processed AZ63 sheet were studied through electron microscopic analysis. The correlation between the mechanical properties, the microstructure, and the ARB processing parameters of an AZ63 sheet were presented. The experimental results have demonstrated that the average grain size of AZ63 Mg alloy processed by ARB was remarkably refined from 12.8 μm to 5.7 μm when the ARB processing temperature was set to 623 K, indicating the occurrence and development of dynamic recrystallization (DRX) nucleation. With the increase in ARB passes, the microstructure obviously became uniform. However, after five passes of the ARB process at 623 K, grains with different crystallographic orientations at the interface can be rearranged to generate the coherent eutectic plane, which inhibits the further refinement of grain size. During the ARB process of the AZ63 Mg alloy, the grain refinement was controlled by twin-induced recrystallization and dynamic recrystallization. Microcracks at the bonded interface of the ARB_1_ sample were eliminated during the following 3~5 rolling passes at 623 K. After three passes of the ARB process at 623 K, the strength and elongation of the AZ63 Mg alloy increased from 232 MPa and 18.5% to 282 MPa and 26.3%, respectively. The tensile fracture morphology of the sample processed by three passes of ARB exhibited numerous dimples, and the slip lines caused by the cooperative deformation of refined grains can produce a network-like dimple structure, indicating that excellent ductile fracture characteristics could be obtained.

## 1. Introduction

Generally, the magnesium alloys have the merits of low density and high specific strength, therefore, they exhibit broad application prospects and development potentials in the fields of aerospace, automotive, and other lightweight manufacturing industries [1]. Nevertheless, the Mg alloys with a hexagonal close-packed (HCP) crystal structure have a limited slip system at room temperature, and thereby the unacceptable plasticity and toughness are usually regarded as the key issues restricting their further large-scale industrial application [2]. The severe plastic deformation (SPD) technique can change the degree of deformation and control the microstructure evolution through repeated deformation with multiple passes, while the shape and dimension of the material can remain unchanged to improve the strength, plasticity, and toughness [3]. Such a technique can also be a novel strategy for improving the mechanical properties of Mg alloys. Some traditional SPD methods include accumulative roll bonding (ARB), equal channel angular pressing (ECAP), cyclic extrusion compression or reciprocal extrusion (CEC), and high-pressure torsion (HPT), as well as multi-direction forging (MDF) [4]. It should be noted that the production equipment used for the accumulative rolling process is facile and suitable for the manufacture of large-size metallic sheets. Meanwhile, the related processing mode can be considered as the most promising method to achieve the continuous and large-scale industrial production of fine-grained or ultrafine-grained materials, and, thus, it has been already received wide concern.

The ARB technique has been extensively applied in the fabrication of high-performance aluminum alloys [5,6,7,8], copper alloys [9], zirconium alloys [10], and various types of composite materials [11,12,13,14,15]. Up until now, the studies on ARB-processed Mg alloy sheets have mainly concentrated on microstructure evolution and the improvement of its macroscopic mechanical properties. For example, Del Valle et al. [16] carried out the ARB process of the AZ61 Mg alloy with reduction rates of 25%, 50%, 66%, and 80%, at 573 K and 673 K, respectively. The corresponding results showed that both the homogenization of the microstructure and the improvement of the interface bonding strength were mainly dependent on the relatively high rolling temperature and large deformation passes, while a more significant microstructure refinement can be achieved at a lower temperature. Moreover, when the AZ61 Mg alloy was subjected to the two passes of the ARB process at 673 K, the microstructure was composed of coarse grains and numerous newly recrystallized grains, and, eventually, the grains could be refined to ~3 μm. This indicated that the microstructure heterogeneity was mainly derived from the rotational dynamic recrystallization (RDR) mechanism during the deformation process. Additionally, Perez-Prado et al. [17,18] investigated the microstructure refinement of the Mg alloy under the processes of large deformation hot rolling and accumulative rolling. When the first pass of rolling was performed on the AZ31 Mg alloy, plenty of equiaxed grains with an average size of 4.2 μm were obtained; however, there was no significant microstructure refinement after the subsequent cyclic deformation. The grain size remained at ~3 μm, and the occurrence of grain refinement was mainly detected during the first pass of the ARB process. This suggested that there was a critical minimum grain size for microstructure refinement, and, thus, the grains cannot be further refined by the subsequent accumulative strains once the critical size has been achieved. Li et al. [19] selected the ME20 Mg alloy as their research object and carried out a sum of eight passes of accumulative rolling to investigate the microstructure evolution and mechanical properties. It was found that numerous dynamic recrystallized grains occurred after the first two passes of the ARB process, and the subsequent deformation could not produce further microstructure refinement, but the microstructure became obviously uniform. After eight passes of accumulative deformation, the microstructure was mainly composed of equiaxed ultrafine grains, and almost no coarse deformed grains were observed. With the increase in rolling passes, the strength of the as-deformed sheet increased while the plasticity decreased, which was mainly caused by the change in texture intensity and microstructure evolution during the deformation process. Zhan et al. [20] obtained the AZ31 Mg alloy sheet with ultrafine grains of 1.3 μm through the accumulative rolling process, and after three passes of accumulative deformation, the microstructure was composed of uniform recrystallized grains; however, the generation of deformation twins was not found. Apparently, dynamic recrystallization can be promoted by the accumulation of strains during the ARB process, leading to the gradual transformation from small-angle grain boundaries to large-angle grain boundaries. Furthermore, the complex interface and shear strain distribution also played crucial roles in the grain refinement process. Wei et al. [21] studied the interfacial microstructure and the mechanical properties of a Mg-14Li-3Al-2Gd sheet fabricated by the ARB process of four sheet layers. It should be pointed out that, with the increase in rolling passes, the interface bonding strength of the ARB-processed sheet increased, whereas the plasticity decreased, which was related to the grain refinement along the vertical rolling direction. Trojanova et al. [22] explored the changes in texture and tensile mechanical properties of the bonding zone of the AZ31 Mg alloy during the accumulative rolling process. It was reported that the anisotropy of the AZ31 Mg sheet decreased with the increase in rolling pass and deformation temperature, and the grain refinement and texture intensity became the key influencing factors. Furthermore, Tayyebi et al. [23] fabricated Al/Cu/Mg multilayered composites via the ARB process combined with annealing. By adjusting the rolling cycle or heat treatment condition, the interfacial microstructure and grain structure of the composites could be controlled properly.

As mentioned above, the ARB process can break through the bottleneck of limited total strain in the thickness direction of traditional rolled materials, and the shape and dimension of the as-prepared metallic sheet can remain unchanged, which also meets the demands of large deformation of materials [24,25]. After the ARB process, the interfaces between various metallic layers should be presented. Meanwhile, the elimination of interfaces is considered to be a sign of the complete metallurgical bonding of ARB sheets, which plays an essential role in improving the mechanical properties of ARB sheets. The AZ63 alloy is a type of Mg–Al–Zn alloy that shows good mechanical properties, corrosion resistance, and electromagnetic shielding performance; furthermore, its mechanical properties can be improved by refining grains on the basis of repeated deformation. As a result, by using the ARB process, the AZ63 alloy can be used to manufacture functional components with good structural strength, which are urgently required by the aviation and electronics industries. Notably, the microstructure evolution of the bonding zone in the ARB process of high-strength steel sheets has been already studied by Seleznev et al. [26]. Due to the differences in the crystal structures and microstructure evolution between steel and Mg alloys, the interface bonding mechanism of ARB-processed Mg alloy sheets is required to be elucidated. However, an in-depth insight into the formation and development process of the interface bonding of ARB-processed Mg alloy sheets is lacking. Moreover, the related mechanisms of interface bonding and the correlation between the interface and the fracture behavior of ARB-processed Mg alloy sheets have still not been clearly clarified, which has limited the further development of the industrial application of this light-weight alloy.

The purpose of this present work is to reveal the influences of ARB parameters on the interface bonding and fracture behavior of an AZ63 Mg alloy sheet, and the adjustment method of the ARB processing parameters of the AZ63 sheet is also explored in order to realize the enhancement of the mechanical properties. Specifically, the accumulative rolling process was performed on the AZ63 Mg alloy, and the resultant microstructure evolution obtained at different rolling temperatures and passes was systematically investigated. The interface bonding behaviors under various ARB processing parameters were analyzed, and the bonding mechanisms of the metallic interface were revealed. Meanwhile, the fracture performances of the AZ63 Mg sheet under various ARB processing parameters were also studied. It is anticipated that this work can provide a theoretical basis for improving the ultimate tensile strength and reasonable ductility of an AZ63 Mg alloy sheet by the ARB process.

## 2. Experimental Section

### 2.1. Materials

**Figure 1:**
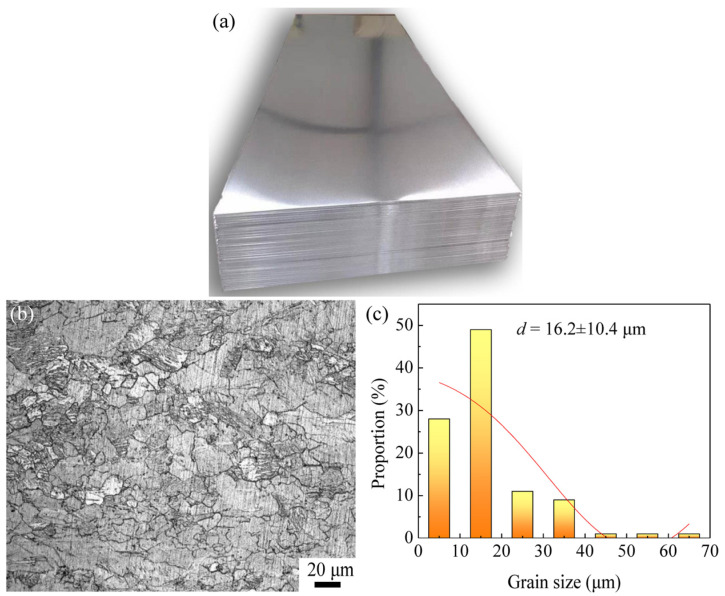
Initial AZ63 magnesium sheet (as-rolled): (**a**) physical image, (**b**) microstructure, and (**c**) grain size distribution.

In this work, the original billet was the commercial AZ63 Mg alloy rolled sheet with a thickness of 2 mm, and it was cut into plates with a size of 60 mm × 100 mm, as shown in Figure 1a. Table 1 lists the chemical composition of the AZ63 Mg alloy. As shown in Figure 1b, the microstructure of the as-received plate is composed of coarse deformed grains with heterogeneous distribution, and the average grain size (*d*) can be determined as 16.2 μm.

### 2.2. Principle of Material Processing

**Figure 2:**
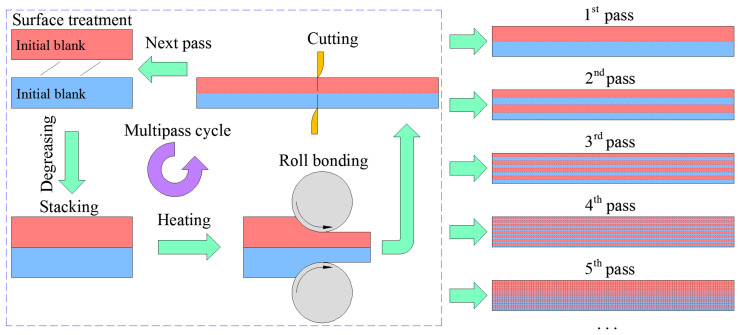
Schematic diagram of the working procedures of the ARB process.

Figure 2 illustrates a schematic diagram of the accumulative roll bonding (ARB) process. It can be divided into five working procedures, including surface treatment, stacking, heating treatment, rolling process, and cutting. During one processing cycle (i.e., one pass), the reduction rate of the sheet was set as a constant value of 50%. The width variation of the sheet can be neglected during the deformation process, and then the macroscopic dimension of the sheet can remain unchanged. Two sheets, obtained after one pass of the ARB process, were restacked together for the next rolling pass. Theoretically, infinite accumulative strains can be imposed on the metallic materials to break through the limitation of the traditional rolling process; therefore, this technique has significant advantages in the continuous production of thin metallic sheets with a refined microstructure and optimized mechanical properties.

During the ARB process, the sample deformed by the first ARB pass was denoted as ARB_1_. Subsequently, the ARB_1_ sheet was cut into two smaller sheets of the same size, perpendicular to the rolling direction, and then the previous process was repeated. The Mg alloy sheets produced by subsequent different cycles were denoted as ARB_2_, ARB_3_, ARB_4_, and ARB_5_, respectively. Table 2 lists the ARB processing parameters used in this study.

### 2.3. Experimental Schemes

First, the surface treatment (i.e., mechanical polishing) was conducted on the samples with the same shape and dimension (i.e., 60 mm × 100 mm × 2 mm) to remove the impurities, and then the surfaces of the samples were cleaned with acetone to remove the oil stains and metal debris so that the bonding strength between the sheets could be enhanced during the rolling process. Secondly, the above surface-treated samples were stacked together and fixed using spot welding to prevent the occurrence of cracking at the front end of the rolling process. After that, the as-stacked sheets were placed in the heating furnace and heated to 523 K, 573 K, 623 K, and 673 K, respectively. In order to ensure the uniform heating of the samples, the heat treatment was maintained for a certain time (15~30 min). Simultaneously, the roller was heated to 423 K, and the processing parameters were set as follows: the rolling speed was 18 r/min, the diameter of the roll was 180 mm, and the deformation was 50%. The combination of rolling force and friction between the sheets can make the two original sheets bond into a single sheet, and the rolls were not lubricated during the ARB process. Due to the imposed intensive deformation, a few cracks on the edge of sample were detected after the first pass of rolling. In order to ensure the quality of the following ARB process, the cracks around the sample were cut off with a shearing machine.

**Figure 3:**
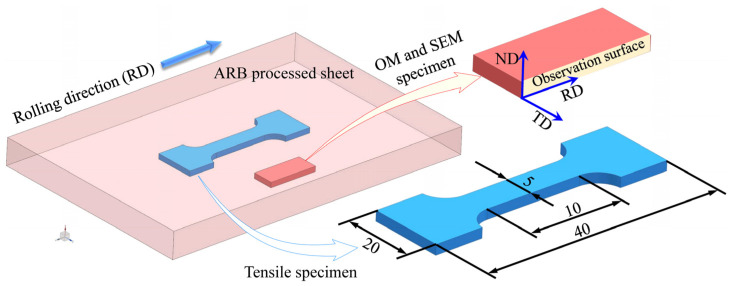
Observation surface of OM and SEM specimens and dimensional drawing of tensile specimen.

The samples were ground on abrasive papers with particle sizes of 800#, 1000#, and 2000#, and then mechanically polished with a polishing machine. After polishing, each sample was etched for around 15 s in the picric acid solution, which contained 5 g of picric acid, 5 mL of glacial acetic acid, 100 mL of absolute ethanol, and 10 mL of distilled water. Subsequently, each sample was cleaned with anhydrous ethanol and quickly dried. Afterwards, the microstructure of each sample was observed with an OLYMPUS-PME3 metallographic microscope. As shown in Figure 3, the RD–ND plane was selected as the observation surface (ND represents the normal direction of the sheet rolling plane and RD represents the rolling direction). Furthermore, scanning electron microscopy (SEM) was also conducted to observe the microstructure characteristics of the ARB-processed samples.

The mechanical properties of the accumulative rolled Mg alloy sheets were evaluated using the uniaxial tensile test on the universal tensile testing machine (model Instron-5948R), with a tensile speed of 0.5 mm/min. A total of 3~5 samples were tensile tested to ensure the reliability of the tensile curves in a single processing condition. The tensile samples were cut off from the center of the ARB-processed Mg alloy sheet along the rolling direction (RD), and the thickness was consistent with the sheet thickness, i.e., 2 mm. The specific sample size is presented in Figure 3. The fracture morphologies of the samples after the tensile tests were also observed with SEM.

## 3. Results and Discussion

### 3.1. Effects of Rolling Temperature on the Microstructure of the ARB-Processed Sheet

**Figure 4:**
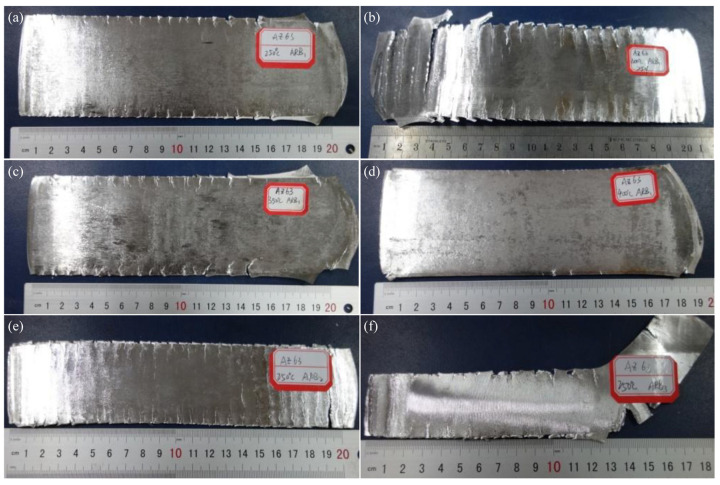
ARB-processed samples after the first pass of rolling (i.e., ARB_1_) at (**a**) 523 K, (**b**) 573 K, (**c**) 623 K, and (**d**) 673 K; and (**e**) the second (i.e., ARB_2_) and (**f**) third (i.e., ARB_3_) rolling passes at 523 K.

Figure 4 shows the macroscopic morphologies of the AZ63 Mg alloy after the first pass of rolling at various temperatures. When the temperature of the ARB process is 523 K, there are only a few cracks on the surface of the sheet during the first pass of rolling. In contrast, numerous cracks can be detected on the edge of the sheet in the second and third pass of the ARB process, and even the surface of the sheet is crossed by some cracks (Figure 4e). However, when the ARB process is performed at the temperatures of 623 K and 673 K, respectively, the stacking effect of the rolling becomes more prominent, and, thus, the rolling performance can be improved. It should be mentioned that the ARB-processed sheet after multi-pass stacking still maintains the excellent features of a smooth surface and edge, a high surface finish, limited cracks, and the macroscopical interface bonding quality.

**Figure 5:**
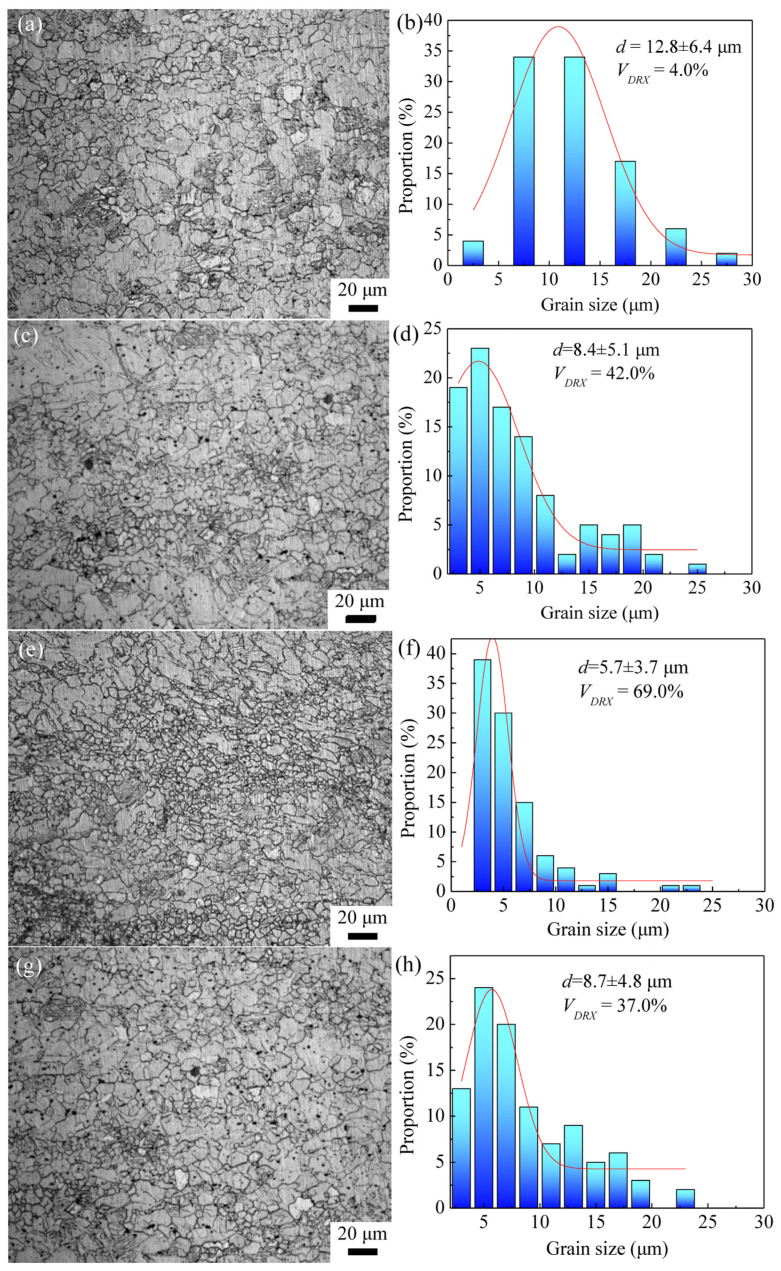
Microstructure and grain size distribution of AZ63 Mg alloy after one pass of ARB deformation (i.e., ARB1) at (**a**,**b**) 523 K, (**c**,**d**) 573 K, (**e**,**f**) 623 K, and (**g**,**h**) 673 K.

Figure 5 shows the OM images and grain size distribution of the ARB_l_ samples with a reduction rate of 50% at temperatures of 523 K, 573 K, 623 K, and 673 K, respectively. The average grain size (*d*) and fraction of the dynamic recrystallization (*V_DRX_*) are presented in the figure. When the temperature of the ARB_1_ process is set at 523 K, plenty of dynamic recrystallized grains can be induced by the introduction of severe plastic deformation, whereas some coarse deformed grains remain, and deformation twins are also generated. The average grain size is around 12.8 μm. It can be seen that the microstructure is composed of coarse deformed grains, deformation twins, and fine dynamic recrystallized grains, indicating that a microstructure with high inhomogeneity is obtained under the experimental conditions of the ARB_1_ process at 523 K. Such observations are similar to those of the AZ31 Mg alloy deformed by two-cycle ARB processing, in which the layered bimodal structures are characterized by an alternative distribution of fine-grained layers and coarse-grained layers [27]. With the increase in temperature, the proportion of coarse grains increases obviously, and the average grain size gradually decreases to 5.7 μm at 623 K, suggesting that the microstructure gradually becomes uniform. When the processing temperature is increased to 673 K, the average grain size shows an increase to about 8.7 μm, and there are several coarse deformed grains. As the same rolling passes of the ARB process are applied, a low deformation temperature can induce a relatively small deformation rate. The work hardening can enhance the driven force of nucleation of the dynamic recrystallization within the coarse deformed grains, leading to the generation of grain refinement. With the increase in temperature, the thermal motion of the solute atoms in the alloy is promoted and the atomic diffusion rate increases. Meanwhile, it can be deduced that the dislocation accumulation is also aggravated to improve the dynamic recrystallization nucleation so that the new equiaxed recrystallized grains can be generated (Figure 5e,g).

### 3.2. Effects of Rolling Passes on Microstructure of the ARB-Processed Sheet

**Figure 6:**
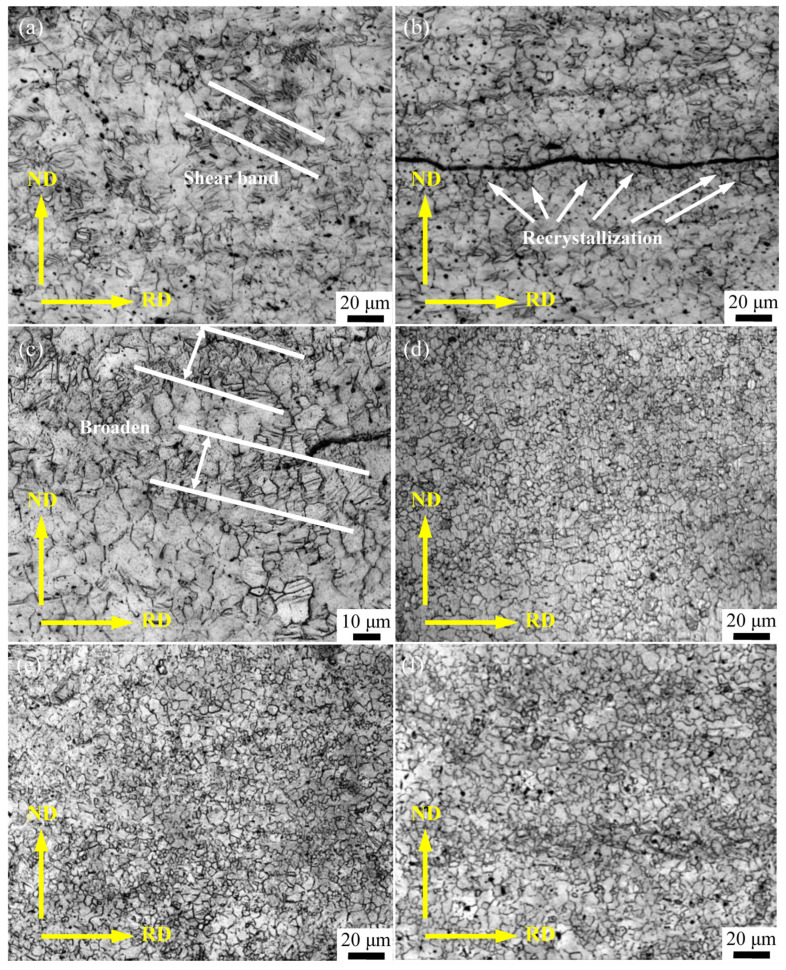
Microstructure evolution of AZ63 Mg alloy after different ARB passes at 573 K: (**a**) and (**b**) ARB_1_, (**c**) ARB_2_, (**d**) ARB_3_, (**e**) ARB_4_, and (**f**) ARB_5_.

Figure 6 shows the microstructure evolution when the ARB process is conducted at 573 K. It can be seen that, with the increase in ARB passes, the microstructure is gradually refined. After the first ARB pass, a large number of deformation twins are generated to subdivide the coarse grains, indicating that refined grains are formed instead of the initial deformed microstructure. Meanwhile, it has also been found that shear bands of 30°–40° with the rolled plane are dispersed within the microstructure (Figure 6a). During the ARB process, a large amount of shear stress can be produced at the interface, which is favorable for the occurrence of dynamic recrystallization; therefore, the size of the grains around the interface is relatively small (Figure 6b).

For the ARB_2_ sample, new shear bands are constantly formed, and their width gradually increases (Figure 6c). Within the shear bands, the deformation twins and fine grains are more densely distributed, and the volume fraction of the deformation twins increases remarkably. When three passes of the ARB process are performed, the microstructure gradually becomes uniform with the accumulation of strains, and thereby the shear bands disappear. As presented in Figure 6e, the microstructure of the ARB_4_ sample is mainly composed of dynamic recrystallized grains with a volume fraction of 92%, and the average grain size can be determined as 4.2 μm. However, the grains within the ARB_5_ sample cannot be further refined, whereas the average grain size becomes relatively larger compared to that of the ARB_4_ sample. This can be explained by the fact that, as the storage energy of the deformation increase during the ARB process, the activation energy of recrystallization nucleation is significantly enhanced, leading to the increased degree of dynamic recrystallization. The initial coarse grains are gradually subdivided and refined by the formation of deformation twins and shear bands, which can be replayed by the dynamic recrystallized grains. The resultant microstructure with fine equiaxed grains (ARB_3_) is presented in Figure 6d.

### 3.3. Interfacial Bonding Behaviors of the ARB-Processed Sheet

**Figure 7:**
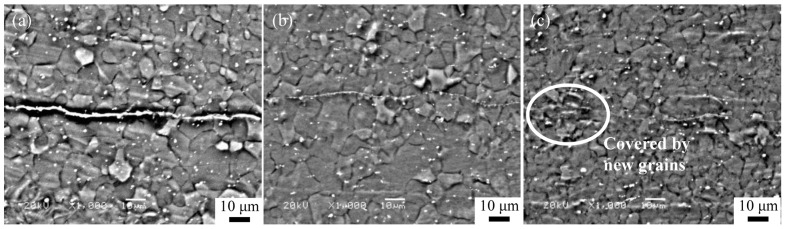
SEM images of the bonded interface of the AZ63 Mg sheet subjected to (**a**) 40%, (**b**) 50%, and (**c**) 60% compression in one ARB pass.

In order to explore the interface bonding characteristics under different deformation strains, microstructure observations were conducted near the interfaces of different samples, as shown in Figure 7. The experimental conditions can be described as follows: the heating temperature was set as 573 K, the holding time was 30 min, the rolls were heated to 423 K, and rolling reduction rates of 40%, 50%, and 60% were applied on the AZ63 Mg alloy. As shown in Figure 7a, a coarse microstructure can be observed when the reduction rate is 40%, and obvious flat interfaces with inclusions or oxide particles can be detected. The thickness of the interface was determined to be about 420 nm, therefore, it is difficult to identify whether the metallurgical bonding can be achieved at the interface. As the deformation amount is increased to 50%, numerous recrystallized grains are formed around the interface (as shown in Figure 7b). It is apparent that the bonding quality of the interface is significantly improved, and the thickness of the interface is reduced to about 190 nm. It can be seen from Figure 7c that the previously remaining coarse grains are replaced by fine dynamic recrystallized grains at the deformation amount of 60%, suggesting that significant microstructure refinement can be obtained, leading to an improved quality of the bonding surface.

**Figure 8:**
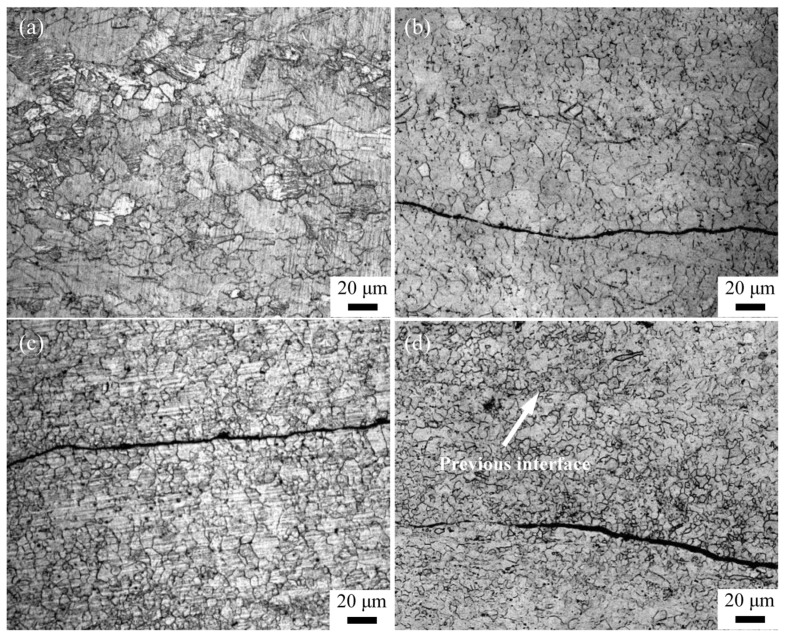
Microstructure of AZ63 Mg alloy in (**a**) initial state and after (**b**) one, (**c**) three, and (**d**) five passes of the ARB process at 623 K.

Figure 8 shows the microstructure characteristics near the interface of the AZ63 Mg alloy after five passes of the ARB process at 623 K. The microstructure evolution of the interface can be clearly observed. The microstructure of the as-received rolled sheet is composed of coarse deformed grains and a band-shaped structure, as shown in Figure 8a. For the ARB_1_ sample, the occurrence of dynamic recrystallization is induced by the imposed large strains, and a small amount of deformation twins are generated. It has also been found that the refined grains are distributed along the obvious hot-compression interface (as marked by the irregular black band in Figure 8b). As shown in Figure 8c, the microstructure is further refined after three passes of the ARB process, and only a small number of coarse grains remain, resulting in an improved uniformity of the microstructure. Moreover, the recrystallized grains are densely distributed around the interface; however, the interface formed by the last pass of the ARB process still exhibits a certain width. For the ARB_5_ sample, the degree of dynamic recrystallization increases with the continuous increase in strain accumulation. In order to further coordinate the plastic deformation without the appearance of cracks, new equiaxed grains are formed around the interface after the last pass of the ARB process, and thereby the uniformity of the microstructure is improved, resulting in an improved interface bonding quality (as marked by the arrow in Figure 8d).

**Figure 9:**
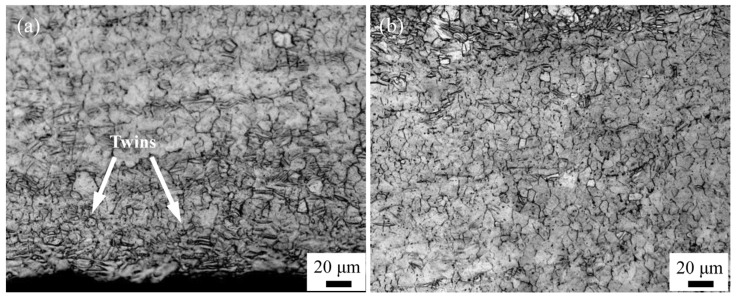
Microstructure of the AZ63 Mg alloy close to the sheet/roller contact surface after (**a**) one and (**b**) three passes of the ARB process at 623 K.

When the sample is subjected to a deformation amount of 50% during the first pass, the shear strains imposed on the surface are sharply increased by the friction with the rolls. Meanwhile, as the distance becomes closer to the surface area, the shear strains become larger, but gradually weakened along the thickness direction of the sheet. Accordingly, as shown in Figure 9a, plenty of deformation twins are generated within the microstructure of the surface area where the sheet and rolls make contact. By comparison, the amount of deformation twins decreases within the microstructure of the central area of the sheet. During the ARB process, the amount of interface also increases as the rolling pass increases. This means that the presence of an oxide film, or a hardened layer on the previous surface, can be broken up into small particles when a new interface is introduced by the following rolling pass, indicating that the grain growth can be suppressed by the accumulated particles. Additionally, the formation of deformation twins on the surface during the previous rolling pass can also act as heterogeneous nucleation sites to promote the occurrence of dynamic recrystallization, resulting in the continuous generation of grain refinement. After three passes of the ARB process, a few of deformation twins still remain within the microstructure of the surface area, whereas the distribution of shear force becomes uniform due to the introduction of multi-layer interfaces, leading to the microstructure refinement of the interior zone of the sheet (Figure 9b). Therefore, the microstructural optimization and grain refinement of the ARB-processed material are derived from the multiple deformation mechanisms.

### 3.4. Twin-Induced Recrystallization and Grain Refinement during the ARB Process

**Figure 10:**
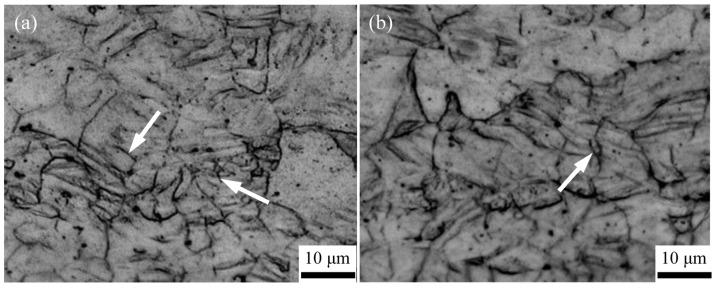
Twin-induced dynamic recrystallization nucleation in the ARB_2_ sample: (**a**) location 1 and (**b**) location 2.

It is well known that, during the plastic deformation of Mg alloys, the deformation twins play a critical role in the dynamic recrystallization nucleation. Figure 10 shows the SEM images of the area enriched with the deformation twins of the ARB_2_ sample processed at 573 K. The dynamic recrystallization nucleation process can be roughly described as follows. The intersections of deformation twins are favorable for the multiplication and accumulation of dislocation, leading to stress concentration. Subgrains with low-angle grain boundaries are generated at the intersections of the deformation twins, and the increase in deformation storage energy provides a sufficient driving force for such nucleation. After that, the initial boundaries of the deformation twins are gradually eliminated by the expansion of the grain boundaries. Thus, the intersections of the deformation twins having sufficient deformation storage energy can provide enough effective recrystallization nucleation sites, and, eventually, new recrystallized grains are formed. In terms of the nucleation mechanism, the formation of deformation twins can induce dynamic recovery and recrystallization, and thereby the grains of shear bands are significantly refined, resulting in an improved plastic deformation ability, to a certain extent.

**Figure 11:**
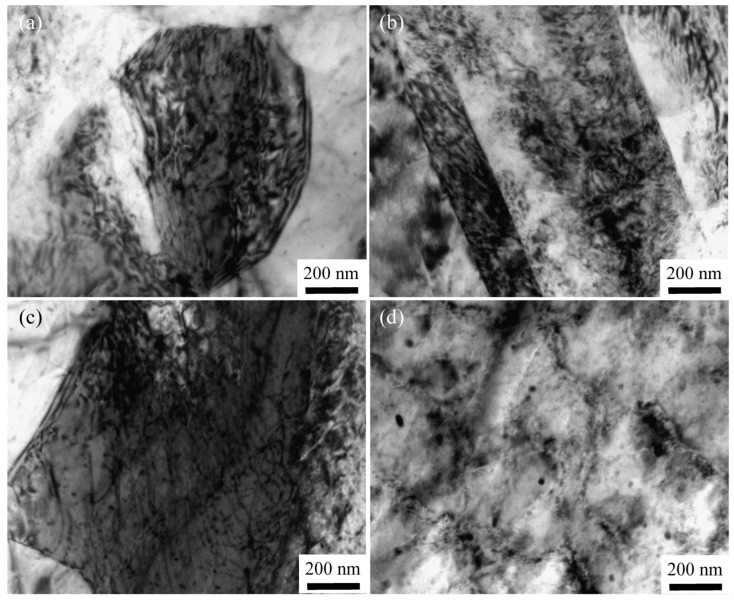
Evolution of dislocations within the grain interior of the ARB_2_ sample: (**a**) in a deformed grain, (**b**) near twin boundary, (**c**) dislocation tangle, and (**d**) dislocation cell.

The grain refinement of the ARB-processed AZ63 Mg alloy can be controlled by twin-induced dynamic recrystallization. In order to further reveal the microstructure refinement mechanism, the dislocation distribution within the grains of the ARB_2_ sample was selected as a representative example, and the TEM images are shown in Figure 11. As shown in Figure 11a,b, numerous dislocations are concentrated within the grain interior and twin boundary, especially at the coarse grain boundary with poor strain coordination, and thereby the dislocations are accumulated and tangled during the ARB process (Figure 11c). Subsequently, the formation of dislocation cells/pileups is promoted by further interactions between the dislocations. Accordingly, the presence of dislocation cells can be detected within the coarse grains, and the dislocation cells are gradually transformed into subgrains when large deformation strains are applied (Figure 11d). As the deformation degree continues to increase, the deformation storage energy can provide an additional driving force for new recrystallized grains.

### 3.5. Fracture Behaviors of the ARB-Processed Sheet in Uniaxial Tensile Stress State

**Figure 12:**
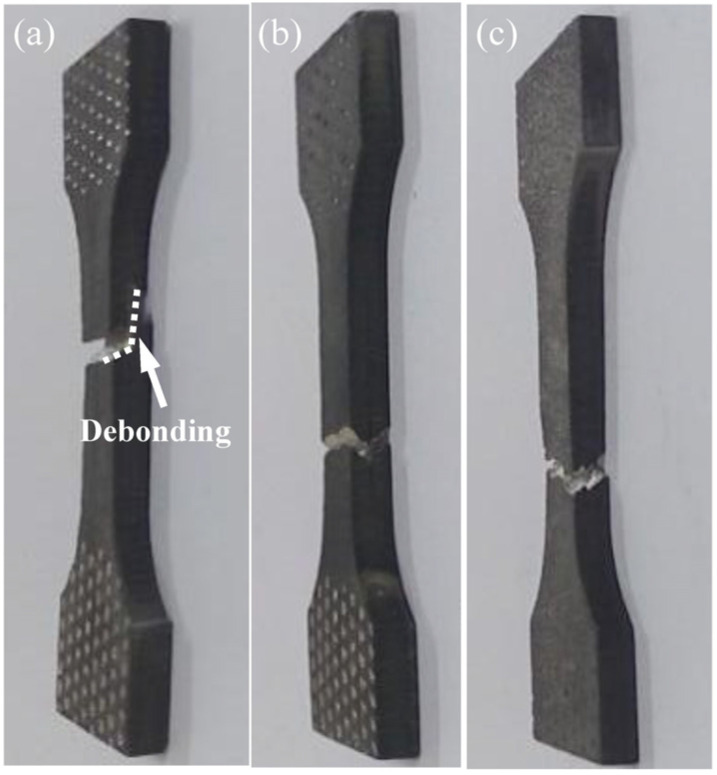
Macroscopic photographs of fractured AZ63 specimens after one rolling pass of the ARB process with (**a**) 40%, (**b**) 50%, and (**c**) 60% deformation.

In order to examine the interface bonding quality and bonding strength, the tensile test was performed on three groups of ARB_1_ samples, and the resultant macroscopic photographs after fracture are given in Figure 12. Figure 12a shows the macroscopic morphology after tensile fracture with 40% deformation. It can be seen that the fracture morphology is clearly delaminated during the tensile test, indicating that the interface cannot be stably bonded. As shown in Figure 12b, when the deformation amount reaches 50%, the occurrence of tensile fracture can be still found at the interfaces. When the deformation amount is increased to 60% (Figure 12c), the macroscopic morphology of tensile fracture is relatively flat, without any obvious delamination.

**Figure 13:**
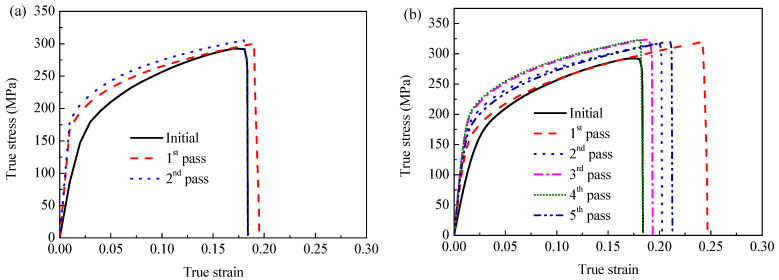

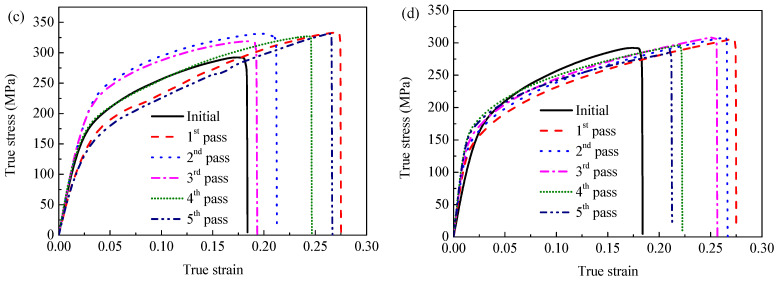
True stress–strain curves of AZ63 Mg specimens processed at different rolling temperatures: (**a**) 523 K, (**b**) 573 K, (**c**) 623 K, and (**d**) 673 K.

**Figure 14:**
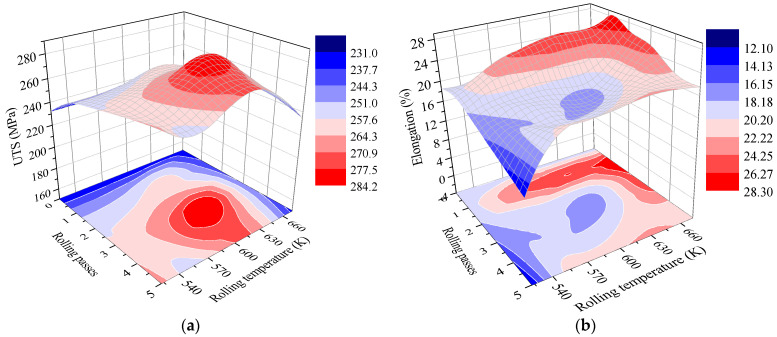
Variation of (**a**) ultimate tensile strength and (**b**) elongation of the ARB-processed AZ63 Mg specimens with the number of rolling passes and rolling temperatures.

True stress–strain curves of the AZ63 Mg specimens processed at different rolling temperatures are presented in Figure 13. It can be observed that the ultimate tensile strength (UTS) of the AZ63 Mg alloy sheet is increased after ARB processing at 523 K, 573 K, and 623 K. The elongation can be increased as well. However, when processed at 673 K, the UTS tends to decrease slightly, but the elongation in this case is preferable. Concretely, Figure 14a,b shows the variation of UTS and elongation with the different rolling passes and rolling temperatures. In general, the UTS of the ARB sample first increases and then decreases with the increase in rolling pass, as shown in Figure 14a. For example, after the first pass of ARB processing (i.e., ARB_1_) at 623 K, the UTS of the sample increased from 232 MPa to 258 MPa. Also, the UTS of the ARB_3_ sample is larger than that of the ARB_1_ sample, reaching an average value of 270 MPa. It can be deduced that the main strengthening effect is derived from the grain refinement induced by the strain accumulation during the ARB process. Moreover, work hardening results in the increase in dislocation density, which is also responsible for the increase in UTS [28]. Similarly, the UTS of the sample first increases and then decreases with the increase in the rolling temperature. The maximum UTS is achieved at 600 K~630 K. This is related to the large amount of DRX at this temperature range. However, excessive temperature may lead to the growth of grains and may decrease the tensile strength.

The variation of the elongation of the ARB-processed sample is almost in reverse. The elongation of the samples first decreases and then increases with the increase in rolling pass. For example, the elongation of the initial as-rolled sample is 18.5%, and it increases to 26.3% after the first rolling pass at 623 K. After this, the elongation of the ARB-processed sample is gradually reduced with the increase in rolling pass; however, it increases again after four passes of the ARB process, and the elongation of the ARB_5_ sample reaches 22.6%. The increase in elongation could also be attributed to the work hardening and fine grain strengthening effects [28]. When the deformation temperature is less than 493 K, the plastic deformation mechanisms of the Mg alloy are usually limited to base slip and pyramidal twinning [29]. With the increase in temperature, more slip systems can be activated to coordinate the plastic deformation of Mg alloys, and the larger degree of deformation can be accommodated to improve the ductility. When the deformation temperature is increased to the range of 573 K~673 K, the slip system in the Mg alloys can be activated to enhance the deformation ability; therefore, the elongation of the sample is increased to a maximum value after the ARB process at 673 K. It is well known that the mechanical properties of Mg alloys are often strongly dependent on the microstructure characteristics. Based on the typical Hall–Petch relationship, the hardness of the microstructure of coarse grains should be less than that of the microstructure of fine grains [27]. During the uniaxial tensile deformation of the bimodal-structured ARB sample, the imposed strains should be continuous at the interlamellar interfaces, leading to the generation of a strain gradient near these interfaces. The geometrically necessary dislocations (GDNs) can be produced to accommodate the inhomogeneous deformation caused by the strain gradient [27,30]. As reported, the constraint effect between soft and hard layers, together with the high yield stress induced by the fine grains, are favorable for the activation of supplementary slip systems that have high critical resolved shear stress (CRSS) [27,31]. Accordingly, the accumulation and interaction of dislocations can be enhanced by the activation of non-basal slip systems in the ARB-processed Mg alloy, which is conducive to realize the high work hardening and reasonable tensile elongation.

**Figure 15:**
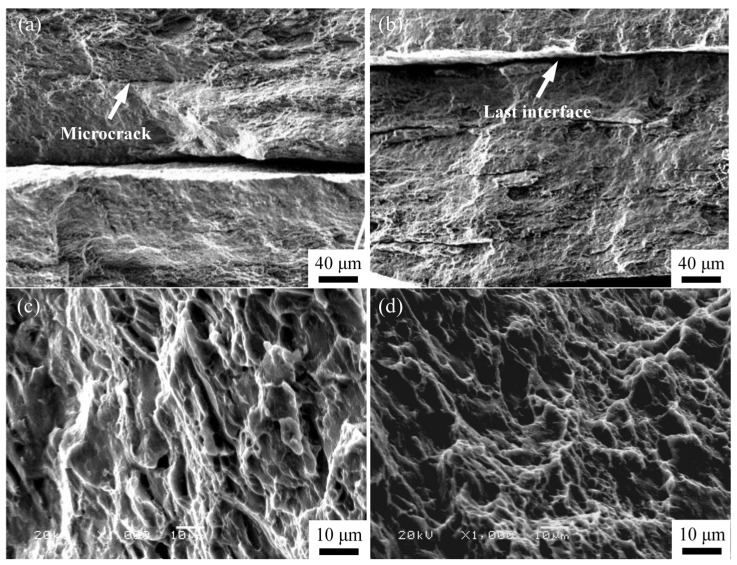
Fracture morphology of AZ63 alloy subjected to (**a**,**c**) one pass (i.e., ARB_1_) and (**b**,**d**) three passes (i.e., ARB_3_) of ARB processing after tensile tests.

In order to further reveal the underlying mechanisms of the interface bonding behavior, the ARB_1_, ARB_3_, and ARB_5_ samples processed at 623 K were selected for the evaluation of their mechanical properties, and the resultant fracture morphologies after the tensile test are given in Figure 14. It was found that the delamination mainly occurs at the interface produced by the final ARB pass, while the previous bonding layer completely disappears. For example, from the macroscopic fracture morphology of the ARB_3_ sample, only the delamination at the final bonded interface can be clearly observed (Figure 15b). There are also small cracks near the interface layer of the ARB_1_ sample (as marked by the arrow in Figure 15a). The uncoordinated deformation of the material during the tensile test is mainly attributed to the inhomogeneity of the microstructure so that the microcracks are formed near the interface (i.e., stress concentration area) of the ARB_1_ sample (Figure 15a). As compared to the ARB_1_ sample, both the microstructure refinement and the uniformity degree of the ARB_3_ sample are improved, whereby the tensile fracture is composed of numerous dimples and slip lines produced by the cooperative deformation between the refine grains. The generation of network-like dimple structures indicates that ductile fracture characteristics have been obtained (Figure 15d).

**Figure 16:**
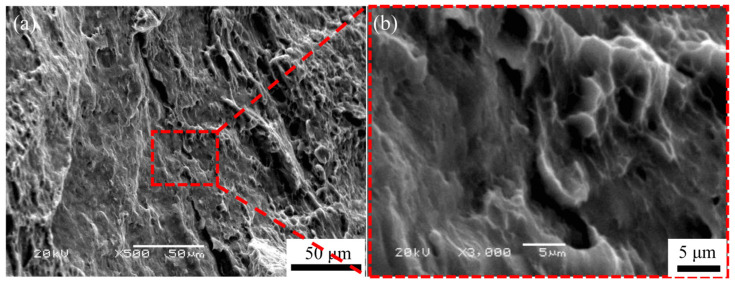
(**a**) Fracture morphology of AZ63 alloy subjected to five passes of ARB processing after tensile tests and (**b**) its enlarged view.

Figure 16a shows the fracture morphology of the bonded interface of the ARB_5_ sample processed at 623 K after the tensile test. Under the action of tensile shear force, the interface exhibits an uneven appearance with a certain degree of non-uniform dimples, which are dominated by shear band tracks and cracks along the rolling direction. Due to the uneven force and uncoordinated deformation of metals at the interface, a shear deformation zone in a “tearing” state can be generated before reaching the ultimate tensile strength of the ARB-processed material. This suggests that, as the coordinate deformation cannot be realized between the interface and the matrix, there are very few uniform dimples, and a fracture eventually occurs along the vicinity of the interface. Figure 16a shows the SEM image of the bonded interface of the ARB_5_ sheet. It was found that the well-bonded interface is composed of dimples after fracture. The ARB_5_ sample exhibits a similar fracture morphology to that of the matrix, as shown in Figure 16b, and no delamination can be detected, indicating that an excellent interface bonding effect can be achieved.

## 4. Conclusions

In this work, AZ63 Mg alloy sheets with excellent mechanical properties were fabricated by the accumulative roll bonding (ARB) process, and the effects of rolling temperature and rolling pass on the interface bonding and fracture behavior of the AZ63 Mg alloy were systematically investigated. The main conclusions can be drawn as follows:

(1) Significant grain refinement of the AZ63 Mg alloy can be obtained by the ARB process, and the corresponding ultimate tensile strength improved. It should be noted that an appropriate increase in rolling temperature can be favorable for the reduced proportion of coarse grains. When the ARB process was conducted at 623 K, the average grain size of the sample after one pass of the ARB process (ARB_1_) reached 5.7 μm. The increase in accumulative strain can induce remarkable grain refinement, and thereby the microstructure uniformity of the sample after three passes of the ARB process (ARB_3_) improved. The ultimate tensile strength of the ARB_3_ sample increased from 232 MPa to 282 MPa, while the elongation of the ARB_1_ sample increased to 26.3%. After this, the elongation gradually decreased with the increase in strain accumulation; however, after five passes of the ARB process, it increased again, and the elongation of the ARB_5_ sample reached 20.3%.

(2) The experimental results demonstrate that the number of rolling passes and the rolling temperatures were the key factors affecting the interface bonding of the AZ63 Mg alloy, and a critical deformation amount (50%) is required in order to obtain high-quality interface bonding. The plastic deformation ability of the Mg alloy can be enhanced by increasing the rolling temperature and prolonging the holding time, which were conducive to the formation of a bonded interface. After five passes of the ARB process at 623 K, the microstructure of the AZ63 Mg alloy evolved from the original coarse deformed grains and twins to uniform recrystallized grains. The bonding quality of the initial interface gradually improved with the subsequent passes of the ARB process. During the ARB process of AZ63 Mg alloy, the grain refinement was controlled by twin-induced recrystallization and dynamic recrystallization.

(3) The microcracks at the bonded interface of the ARB_1_ sample can be eliminated during the following hot rolling at an appropriate temperature, for example, the ARB_5_ sample at 623 K without microcracks. Furthermore, significant microstructure refinement can be detected in the ARB_3_ sample processed at 623 K, indicating that the uniformity of deformation enhanced. The tensile fracture morphology of the ARB_3_ and ARB_5_ samples all consisted of numerous dimples and slip lines formed by the cooperative deformation of the refined grains, and thereby the network-like dimple structure was generated, indicating that good ductile fracture characteristics can be achieved in the AZ63 Mg alloy processed by 3~5 passes of ARB.

However, it must be noted that obvious fluctuation was observed in the elongation and UTS data obtained from tensile tests of the ARB-processed samples. The variations of elongation and UTS with rolling passes and rolling temperatures are not monotonic. This is contradictory to the conventional wisdom and could not be explained in an appropriate theory. Further study on the reason behind this phenomenon is essential. In addition, work hardening is always accompanied by softening at an elevated temperature in hot deformation. However, the influence of work hardening on the microstructure and mechanical properties of an ARB-processed sheet can hardly be determined according to the experimental evidence shown in this work. Since work hardening is also an important mechanism in the analysis of hot deformation, it also deserves further investigation.

## Figures and Tables

**Table 1:** Chemical composition of AZ63 Mg alloy (mass fraction, %).

Elements	Al	Zn	Mn	Si	Cu	Ni	Fe	Mg
Wt.%	6.37	2.96	0.44	0.007	0.005	0.002	0.001	Bal.

**Table 2:** Parameters of sheet metal processed by ARB.

No.	Number of Cycles	Number of Layers	Number of Bonded Boundaries	Layer Interval (μm)	Total Reduction (%)	Equivalent Strain
ARB_1_	1	2	1	500	50	0.8
ARB_2_	2	4	3	250	75	1.6
ARB_3_	3	8	7	125	87.5	2.4
ARB_4_	4	16	15	62.5	93.75	3.2
ARB_5_	5	32	31	31.2	96.875	4.0
	*n*	2*^n^*	2*^n^*− 1	*H*_0_/2*^n^*	(1 − 2*^n^*) × 100	0.8*n*

Note: These parameters were calculated by assuming the initial thickness of the sheet metal as 1 mm.

## Data Availability

The data used in this manuscript can be provided on demand.
